# A Retrospective Analysis of 5,195 Patient Treatment Sessions in an Integrative Veterinary Medicine Service: Patient Characteristics, Presenting Complaints, and Therapeutic Interventions

**DOI:** 10.1155/2015/983621

**Published:** 2015-12-21

**Authors:** Justin Shmalberg, Mushtaq A. Memon

**Affiliations:** ^1^Small Animal Clinical Sciences, College of Veterinary Medicine, University of Florida, Gainesville, FL 32608, USA; ^2^Department of Veterinary Clinical Sciences, College of Veterinary Medicine, Washington State University, Pullman, WA 99164, USA

## Abstract

Integrative veterinary medicine, the combination of complementary and alternative therapies with conventional care, is increasingly prevalent in veterinary practice and a focus of clinical instruction in many academic teaching institutions. However, the presenting complaints, therapeutic modalities, and patient population in an integrative medicine service have not been described. A retrospective analysis of 5,195 integrative patient treatment sessions in a veterinary academic teaching hospital demonstrated that patients most commonly received a combination of therapeutic modalities (39% of all treatment sessions). The 274 patients receiving multiple modalities were most frequently treated for neurologic and orthopedic disease (50.7% versus 49.6% of all presenting complaints, resp.). Older neutered or spayed dogs (mean age = 9.0 years) and Dachshunds were treated more often than expected based on general population statistics. Acupuncture, laser therapy, electroacupuncture, and hydrotherapy were frequently administered (>50% patients). Neurologic patients were more likely to receive acupuncture, electroacupuncture, and therapeutic exercises but less likely than orthopedic patients to receive laser, hydrotherapy, or therapeutic ultrasound treatments (*P* < 0.05). The results suggest that the application of these specific modalities to orthopedic and neurologic diseases should be subjected to increased evidence-based investigations. A review of current knowledge in core areas is presented.

## 1. Introduction

Integrative medicine describes an increasingly popular form of medicine combining conventional medical practice with alternative or complementary therapies, which is based on the best available scientific evidence [1]. Alternative or complementary therapies are broadly defined in human medical practice but may include nutrition, acupuncture, laser therapy, hyperbaric oxygen, rehabilitation, and other interventions not typically considered mainstream medical practice. Integrative* veterinary* medicine is poorly described both in definition and in practice although the term occasionally appears in the scientific literature [2]. However, a similar definition as to that used in integrative human medical practice characterizes the concept in veterinary medicine. Alternate and historical terms used to describe unconventional therapies inadequately distort the purpose of such treatments. Alternative veterinary medicine suggests that certain therapies are a replacement or a mutually exclusive option to conventional care, which disregards the potential for synergistic effects. Complementary medicine implies that the therapies can and should only be used in tandem, when in some cases a modality may be the preferred or exclusive treatment available. Finally, holistic medicine suggests that conventional veterinary practice does not consider the impacts of treatment on the whole animal, an obviously flawed assumption.

The prevalence of integrative medical interventions in veterinary medicine has not been established. A survey of owners of veterinary oncology patients found a robust usage of therapies regarded as alternative or complementary [3]. A survey of one school's veterinary graduates identified that more than two-thirds of these veterinarians encountered clinical situations involving these therapies at least monthly and over 25% experienced them on a weekly or daily basis [4]. These findings served as a framework for that study's authors to suggest, with evidence from surveys of AVMA-accredited colleges of veterinary medicine, that a comprehensive curriculum should be available to veterinary students. The need for education and information in integrative medicine is highlighted by the fact that nearly one-third of the general population has used a complementary or alternative medical approach for their own health [5].

The purpose of this study was to retrospectively evaluate the caseload from within a busy academic integrative veterinary medicine service to determine the frequency with which specific modalities were used and the relationships of such modalities to presenting complaint, breed, age, and other factors. Study findings provide critical information to other integrative medicine services and for researchers investigating specific modalities within the scope of the service's practice. Future randomized controlled trials are needed to further evaluate a number of the modalities.

## 2. Material and Methods

The electronic medical records were collected from a mixed animal integrative medicine service at an academic teaching hospital over a 400-day period from July 2014 to August 2015. The total number of patient visits in each hospital service was tabulated. The presenting complaints for integrative medicine visits were recorded along with the species of patient, body condition score, outpatient or inpatient status, and the date of treatment.

The records of small animal patients receiving more than one therapeutic modality at a visit were further analyzed to determine whether each patient had seen another service in the 6 months before treatment and to calculate the number of integrative visits for each patient. If patients were seen more than once during the retrospective period, a visit was randomly selected to determine which therapeutic modalities the patient received.

The differences between groups were evaluated using one-way ANOVA and commercially available statistical software (Minitab 17.1). Results were considered statistically significant if the probability of error was less than 5% (*P* < 0.05). Post hoc analysis was performed with Fisher's test for pairwise comparisons. Odds ratios were calculated, using commercial software (Microsoft Excel 2010), to assess if specific patient populations, grouped by condition or breed, had a different likelihood of receiving each modality or of presenting with a particular complaint. A result was considered statistically significant if the 95% confidence interval for the odds ratio excluded the value 1.0. The Pearson correlation coefficients (*r*) were calculated for the potential relationships between age, body weight, number of treatments, and the number of modalities used for each patient; results were considered significant if *P* < 0.05.

## 3. Results

**Figure 1:**
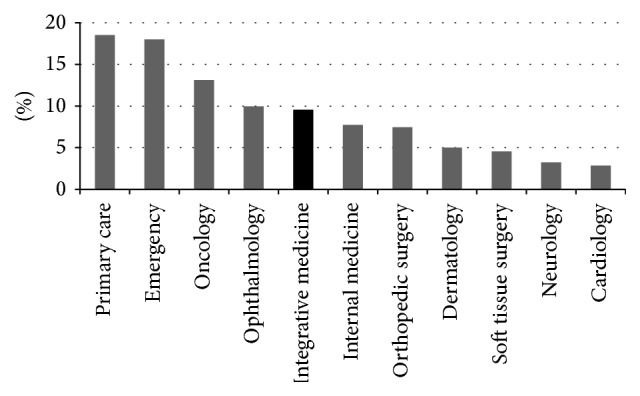
Comparative caseload of the study site's Academic Veterinary Hospital.

The integrative medicine service attended to 5,195 patient treatment sessions during the study period. The distribution of species from greatest to least was as follows: dogs (95.6%), cats (3.0%), horses (0.8%), and exotic species or wildlife (0.6%). The majority of these cases were managed as outpatients (90.2%), with the remainder receiving inpatient treatment (9.8%) over an average inpatient rehabilitation period of 3.0 days. Recorded treatments included those performed during business hours (93.3%) and after hours (6.7%). The integrative medicine service received 9.6% of total hospital caseload (Figure 1).

**Figure 2:**
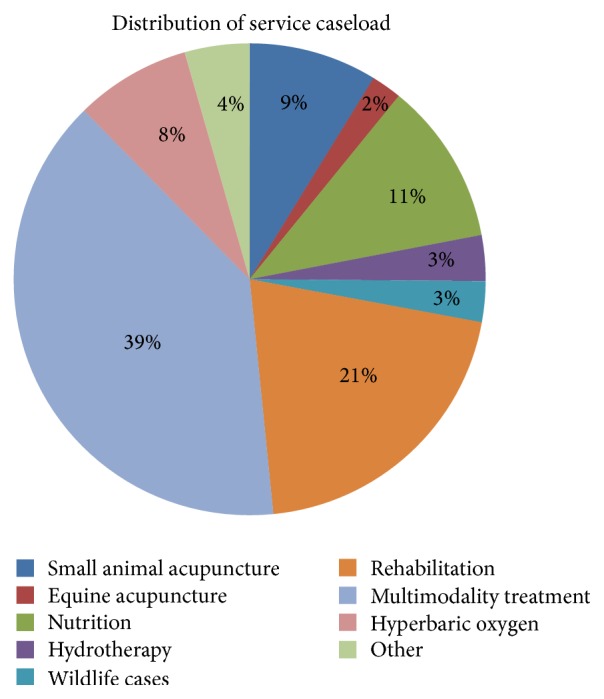
Distribution of integrative medicine patient visits of the study site's Academic Veterinary Hospital.

A number of different therapies are included in the total treatment sessions. Outpatient integrative medicine sessions, which included multiple modalities, were the most prevalent (*n* = 2,042 or 39.3%), followed by rehabilitation-exclusive appointments (20.5%) and nutrition visits (11.1%). The complete distribution of service caseload is provided (Figure 2).

**Figure 3:**
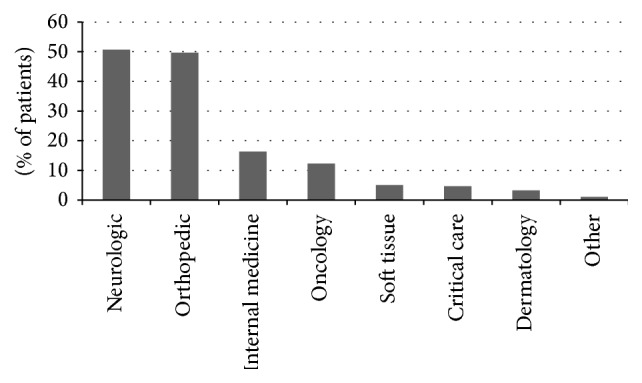
Presenting complaints of veterinary patients receiving multiple therapeutic modalities in the study site's integrative medicine service.

The patients who received multiple therapies were selected for additional analysis. The multiple modality treatment sessions were most commonly utilized for patients (*n* = 274) with neurological and orthopedic conditions (50.7% and 49.6%, resp.). 17.4% of patients presenting with primary neurologic disease had concurrent orthopedic abnormalities, and 15.6% of patients presenting with orthopedic disease had concurrent neurologic disorders. Patients also presented with issues related to internal medicine, oncology, soft tissue surgery, critical care, dermatology, and other conditions (Figure 3). Each patient receiving multiple modalities visited the service an average of 7.6 ± 10.5 times during the study period with a range of 1–106 visits. Orthopedic conditions were treated on average with more visits (10.1 ± 14.2) than were neurologic conditions (6.7 ± 8.9) (*P* = 0.007). The number of visits did not differ by breed (*P* = 0.128) or by age (*P* = 0.68).

**Figure 4:**
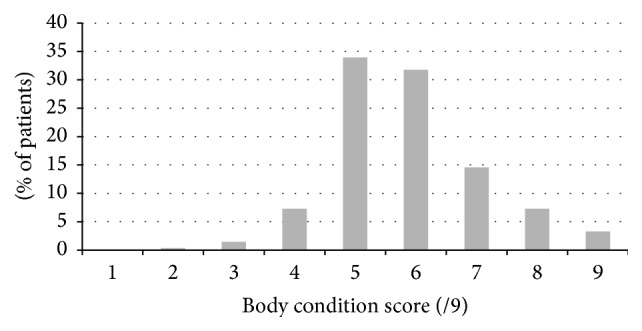
Body condition of veterinary patients receiving multiple integrative therapeutic modalities at the study site's Academic Veterinary Hospital.

The majority of small animal patients that presented for multiple modality visits were of ideal body condition (body condition score (BCS) = 4-5/9, 41.6%) but a significant number were classified as overweight (BCS = 6, 31.8%) or obese (BCS ≥ 7, 25%) (Figure 4). No statistical difference was noted in the body condition scores of those patients presenting for neurologic, orthopedic, or other conditions (*P* = 0.15). A weak negative correlation was observed between the body condition score of the patient and the number of visits (*r* = −0.12, *P* = 0.04).

Patients receiving multiple modalities received 4.1 ± 1.6 different therapies at a visit, with a range of 1–11 modalities. No statistically significant differences were identified in the number of modalities used in patients with neurologic, orthopedic, or other conditions (*P* = 0.19). A weak positive correlation was identified between the number of modalities employed and the number of visits (*r* = 0.21, *P* = 0.001). No differences were detected in the number of modalities any breed category received (*P* = 0.07).

**Figure 5:**
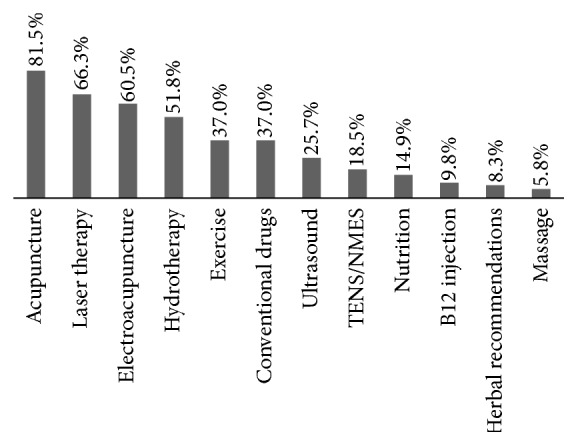
The percentage of patients receiving each integrative therapeutic modality at the study site's integrative medicine service.

Four modalities were used in greater than half of the treatment sessions analyzed. These included acupuncture (81.5% of all treatment sessions), laser therapy (66.3%), electroacupuncture (60.5%), and hydrotherapy (51.8%). Eight other interventions were used for the mixed modality patients but less frequently (Figure 5).

**Figure 6:**
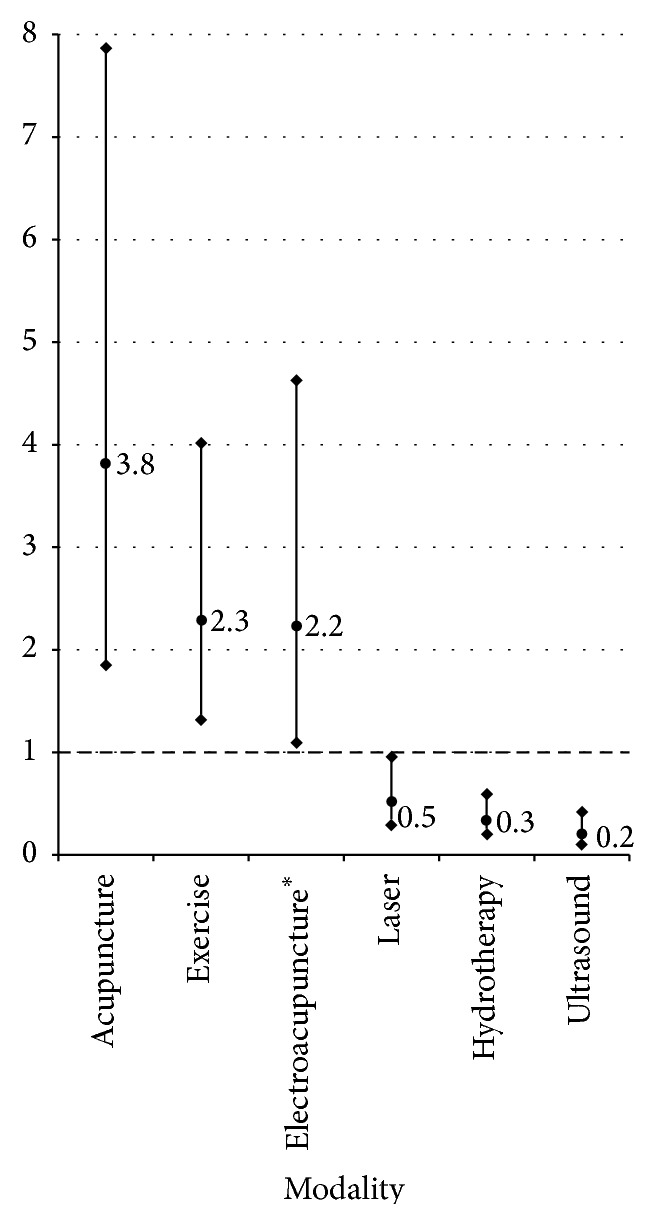
The odds of receiving six modalities were different in neurologic as compared to orthopedic patients (OR, 95% CI) of the veterinary patients at the study site's integrative medicine service. ^*∗*^ The odds ratio was calculated by comparing the odds of neurologic patients with acupuncture receiving electroacupuncture as compared to orthopedic patients with acupuncture receiving electroacupuncture. Electroacupuncture was always performed with acupuncture, and therefore this comparison normalizes for the observed differences in the odds of receiving acupuncture alone.

Neurologic patients were significantly more likely to receive acupuncture, electroacupuncture, and rehabilitation exercises as compared to orthopedic patients (*P* < 0.05, Figure 6). These patients were less likely to receive laser therapy, hydrotherapy, and ultrasound. There was no difference in the odds of either group receiving transcutaneous electrical nerve stimulation (TENS) or neuromuscular electrical stimulation (NMES), nutritional or herbal recommendations, massage, conventional drugs, or cyanocobalamin injections.

Sixty-two breeds of dogs were treated. Mixed breed dogs were most commonly presented (27.0%). Both Dachshunds and Labrador retrievers were overrepresented in the patient population when compared to other breeds, amounting to 15.2% and 7.4% of the study population, respectively. German Shepherds comprised 3.3% of patients, and the remainder of breeds accounted for less than 2.2% of the dogs treated.

**Figure 7:**
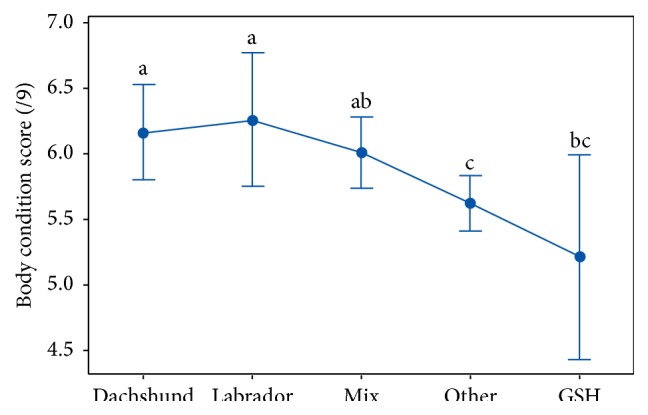
Breed differences in body condition score at the study site's integrative medicine service (breeds with different letters are statistically different).

Analysis of the five major breed categories (Dachshunds, Labrador retrievers, mixed breeds, German Shepherds, and a group of all other breeds) did not show any statistically significant difference in age (*P* = 0.12). Labrador retrievers and German Shepherds were heavier than mixed and other breeds in this study, and all four groups were more massive than Dachshunds. Statistically significant differences were noted between breeds and average body condition scores (*P* = 0.008, Figure 7).

Multiple modality treatments were employed primarily for dogs (*n* = 270) and also for cats (*n* = 4). The mean age of cats was 14.0 years (10–17) with a body weight of 4.6 kg (3.5–5.9). All cats were neutered males. The mean age for dogs (*n* = 270) was 9.0 ± 3.9 years (1–18) with a body weight of 19.2 kg ± 12.8 (1.2–63.2 kg). The genders of dogs were as follows: 23 intact males, 127 neutered males, 11 intact females, and 109 spayed females. The majority of the animals treated were seen either concurrently or within the previous six months by another service in the same academic teaching hospital (92.2%, *n* = 249).

Statistically significant differences were determined between dogs grouped by presenting condition. Dogs presenting for orthopedic conditions weighed less than those presenting for neurologic or for other conditions (14.9 ± 13.1 versus 23.3 ± 11.3 and 19.8 ± 12.5 kg, resp. *P* < 0.001). Dogs were younger if treated for orthopedic as opposed to other conditions (8.3 ± 3.4 years versus 9.8 ± 3.6 years) (*P* = 0.048) but were not different in age from those treated for neurologic diseases. No difference was detected in the body condition scores of dogs when grouped by presenting complaint (*P* = 0.12). There was no difference in the number of modalities received for dogs presenting with orthopedic, neurologic, or other problems (*P* = 0.57).

Dachshunds were substantially more likely to present with a neurologic condition than with orthopedic or other conditions (OR 23.6; 7.47–74.5). Conversely, Labrador retrievers were more likely to be treated for a primary orthopedic complaint as opposed to a neurologic issue (OR 6.0; 1.46–24.7). Predictably, Dachshunds were more likely to present with neurologic conditions than were Labradors (OR 19.4; 4.96–76.1), mixed breed dogs (OR 14.8; 5.61–39.2), or other breeds (OR 7.48; 3.08–18.2). No Dachshunds presented with orthopedic complaints and they were therefore the least likely of the breeds to present with this condition.

The odds ratios for dogs receiving some modalities differed significantly among the breed categories (Dachshunds, Labradors, German Shepherds, mixed breed dogs, and the group of other dog breeds). Dachshunds were less likely to receive B12 injections than were German Shepherds (OR 0.10; 0.01–0.74) and were less likely to receive therapeutic ultrasound than mixed breed dogs (OR 0.21; 0.07–0.60). They were, however, more likely than other breeds to receive TENS/NMES (OR 2.51; 1.08–5.79) and also more likely than mixed breeds (OR 2.98; 1.33–6.68) and Labrador retrievers (OR 5.95; 1.51–23.5) to receive therapeutic exercises. Mixed breeds were more likely to receive therapeutic ultrasound than other breeds (OR 2.44; 1.30–4.60) and less likely to receive B12 injections than were German Shepherds (OR 0.15; 0.04–0.49). Other breeds were less likely than German Shepherds to receive conventional prescription drug recommendations (OR 0.21; 0.05–0.90).

## 4. Discussion

The integrative medicine service from which data was derived is unique in several respects. The academic mission of the veterinary college necessitates a model that incorporates student experiential learning, the training of house officers, and an emphasis on inter-specialty cooperation and on evidence-based medicine. The service was staffed during the study period by 1.25–2.25 full-time-equivalent faculty, 1-2 house officers, and two certified rehabilitation veterinary technicians. Four to five students were present in rotating two-week elective rotations for the entire year. The integrative nature of the service is supported by data in the present investigation; a large number of patients also presented to other hospital services (92%), and a recommendation of conventional therapies was made in 36.6% of multimodality treatment visits. Comparative data from integrative services at other institutions are unavailable, and no other service combines the same therapeutic options. Therefore, additional data and descriptions from other services are required before comparative conclusions can be made.

The service offers four of the five most commonly taught modalities in veterinary schools: nutrition, rehabilitation, acupuncture, and herbal therapy [4]. The notable exception is veterinary spinal manipulative therapy, also referred to as veterinary chiropractic. This practice, based on a principle that manually applied forces induce joint mobility and subsequent myofascial effects, has been objectively studied in horses but not in dogs [6]. Anecdotal concerns about mobilization and worsening of intervertebral discs exist, and practitioners of the modality do advise caution [7]. The absence of evidence-based testing and the lack of training by the service clinicians precluded the use of this technique. Other therapies discussed in the context of integrative medicine were also not a component of the service, including homeopathy, which is derived from foundational principles that “like treats like” and that serial dilutions of a compound increase the potency of a remedy. A recent meta-analysis of veterinary homeopathy found only scant data supporting its use in randomized controlled trials, and the two acceptable studies focused on bovine and porcine treatments [8]. Philosophical arguments surround the plausibility of the therapy, but nevertheless integration into a conventional veterinary hospital is both difficult and questionable given the competing tenets [9, 10].

The study data suggest that multimodality therapies were the most frequent treatment intervention elected by the attending clinicians. The patients receiving a multimodality therapy session were treated in the absence of the owners, from whom a brief history was obtained at the start of the visit and who were updated at the conclusion of the visit. The clients were charged a flat treatment fee irrespective of the number of modalities performed in order to eliminate the appearance of any financial incentive to expand a treatment protocol. Therefore, the patients in this study, which received a range of 1–11 modalities, were all charged equally, and patients on average received four of the available modalities based on clinician assessments. The students were permitted to assist with these patients, and the overall efficiency and case volume was managed by having the patients in the service for several hours.

### 4.1. Acupuncture, Electroacupuncture, and Vitamin B12 Injections

Acupuncture and electroacupuncture were two of the four most frequently administered treatments. Acupuncture remains a source of significant controversy in veterinary medicine. Much of this controversy is derived from debates surrounding the antiquity of acupuncture, and available evidence suggests that modern veterinary acupuncture is a recent invention [11]. Veterinarians may place acupuncture needles based on traditional Chinese practices, on medical explanations and published studies of clinical effects or on a combination of the two approaches [12, 13]. A dated systematic review found there was insufficient evidence to recommend acupuncture in small animal patients like those of the study population [14]. Admittedly, two studies failed to document a significant benefit in dogs with both acute and chronic orthopedic injury [15, 16]. However, studies of varying methodological quality suggested an improvement in neurologic function of dogs affected by intervertebral disc disease when treated with electroacupuncture [17–19]. Interestingly, dogs treated in the retrospective period of this study were more likely to receive acupuncture and electroacupuncture when presenting with neurologic as opposed to orthopedic issues. Some studies of naturally occurring canine pain support an analgesic effect of acupuncture which could be beneficial for many conditions [20, 21]. A number of mechanisms have been postulated for the effects of acupuncture, including endogenous opioid release, substance P modulation, myofascial input, cannabinoid receptor modulation, and other cellular mediators. However, detailed evidence-based discussions should inform the location, effects, and utility of acupoints, several examples of which have been recently published [22, 23].

Cyanocobalamin injections (vitamin B12) were administered infrequently in patients (<10%). The technique has been recommended as an adjunctive method of acupoint stimulation [13], although it has not been scientifically validated. Cobalamin requires intrinsic factor, produced in the stomach and pancreas of dogs and in the pancreas of cats, for intestinal absorption and is a critical cofactor in enzymes for carbon rearrangement and methyl donation [24]. Cyanocobalamin injections have a wide margin of safety, and physiologic and cellular deficiencies have been reported in various conditions [24]. The finding that German Shepherds received B12 injections more commonly than some other breeds is likely unintentional. The fact that oncology patients accounted for the largest number of injections may suggest an intentional supplementation of this group due to perceived neoplasia-related true or relative deficiencies [25]. No animals were tested for their B12 status prior to supplementation so the effectiveness of such an approach remains unclear.

The application of electrical current to a patient's soft tissues is not unique to electroacupuncture. However, electroacupuncture has been shown in multiple species to augment therapeutic response by enhancing the release of endogenous opioids [26]. The best available data suggests that low frequency (2 Hz) acupuncture releases *μ*-acting opioids whereas high frequency stimulation (100 Hz) affects *κ* receptors. Veterinary studies frequently employ a mixed low and high frequency treatment of at least 20-minute duration [15, 17, 20]. Release of *β*-endorphins was documented three hours after treatment in dogs receiving an uncommon protocol of electroacupuncture (24 Hz and 43 Hz) [21]. Horses had a measurable rise of the same opioid in CSF for 2 hours following a 15–30 Hz treatment [27]. The most common protocol used in the present investigation was 2 Hz for 20 minutes in dogs, but additional studies are required to determine the optimal electroacupuncture frequency for the common neurologic and orthopedic conditions encountered during the study period.

### 4.2. TENS and NMES

Transcutaneous electrical nerve stimulation (TENS) utilizes conductive pads to activate sensory nerve fibers and to modulate pain. This therapy was employed in only about 18% of the study patients and was often used in Dachshunds. A recent meta-analysis of human patients found some favorable evidence for TENS treatment of the pain associated with knee osteoarthritis, but subsequent small studies were unable to confirm the effect [28]. TENS treatments set to a frequency of 70 Hz improved ground reaction forces in osteoarthritic dogs [29]. TENS was also employed in a canine physiotherapy program but at unknown settings [30]. TENS units frequently contain a different program for neuromuscular electrical stimulation, which is employed in the treatment of atrophy by recruiting motor fibers. Increased muscle mass was documented in dogs receiving this type of stimulation following surgical repair of a cranial cruciate ligament repair [31]. TENS or NMES could be theorized to be more beneficial for the treatment of large areas given the greater current dispersal area over the pads whereas electroacupuncture might more effectively treat deep tissues; neither suggestion has been scientifically evaluated. Whether the clinical patients in the present retrospective would have benefited more from one therapy as opposed to the other remains unknown.

### 4.3. Laser Therapy (Photobiomodulation)

A therapeutic laser treatment was performed in a majority of the study population. The cellular effects of laser treatments, often referred to as photobiomodulation, are well established and include photonic changes to cytochrome c oxidase resulting in increased cellular energy (ATP) production, the release of nitric oxide, and the generation of free oxygen radicals which stimulates an endogenous antioxidant production [32]. The primary controversy of laser therapy is the dose needed to achieve such biologic effects given that water, melanin, and hemoglobin all absorb photons in a similar spectrum as the target cytochromes. Laser penetration has been poorly studied in dogs, although a study in horses showed consistent differences in penetration in shaved versus unshaved areas [33]. A single clinical trial of canine laser therapy found that dogs had a significantly reduced time to ambulation when treated with laser following a decompressive hemilaminectomy [34]. Therefore, the observation in this study that neurologic patients were less likely to receive laser therapy deserves attention because the strongest clinical evidence supports its use in these patients. Failure to provide this therapy may have been due to pragmatic considerations given that a number of the postoperative patients treated by the service have an adhesive, absorbent, and poorly penetrable bandage over the surgical incision.

### 4.4. Hydrotherapy: Underwater Treadmill Walking and Swimming

Hydrotherapy was more commonly used for orthopedic than neurologic patients, which may be because the service treats a large number of paralyzed dogs with no motor function at the outset of treatment. Neurosurgical patients in the academic teaching hospital typically have intravenous catheters for approximately 48 hours after surgery, and urinary catheters are frequently placed for lower motor neuron bladder dysfunction. The presence of either catheter complicates hydrotherapy, but the degree to which this influenced the odds ratio of hydrotherapy in orthopedic as compared to neurologic patients could not be quantified from the retrospective data available. Additionally, the available evidence for hydrotherapy is presently limited to orthopedic pathology although some authors advocate introduction of the technique 3 to 5 days after surgery for intervertebral disc disease and other neurologic conditions [35]. Underwater treadmill therapy reduces concussive forces on joints and promotes increased joint flexion and full active extension [36]. It has been used for both weight loss protocols and for postoperative rehabilitation of dogs following a tibial plateau leveling osteotomy [37, 38]. Hydrotherapy was defined as either swimming or underwater treadmill therapy in this study, and the exact distribution of each was not collected. Swimming, however, can be used to maximize active range of motion; hip, stifle, and hock flexion were all increased in a pool as compared to a ground treadmill after cranial cruciate ligament repair and in healthy dogs [39]. Conversely, hip and stifle extension angles were reduced by about 13 and 19 degrees, respectively, in swimming dogs after cruciate repair as compared to healthy controls. This was not observed in the same dogs when walking at two speeds on a land (nonaquatic) treadmill [39]. Severely osteoarthritic dogs often exercised more comfortably in a pool because they are completely nonweight bearing. Additional data is required to better inform the optimal time for starting hydrotherapy and the clinical benefits, if any, in neurologic patients.

### 4.5. Therapeutic Exercise

Therapeutic exercises were performed less frequently than hydrotherapy, presumably because the service first exercises patients in water before advancing them to weight-bearing activities with the aid of ground treadmills or other interventions, such as cavaletti poles, weaves, balance ball, and related rehabilitation techniques. Several authors have advocated early limb use after injury in animals, and the kinematics of many techniques are described for dogs [40–43]. However, no study has yet independently associated any one particular exercise with any improvement in clinical outcome. Dachshunds and neurologic patients in the present study were interestingly more likely to receive therapeutic exercises than Labrador retrievers, mixed breeds, and those with orthopedic conditions. This may be due to the fact that many neurologic dogs were paretic and therefore more difficult for owners to exercise when compared to those with orthopedic pathology.

### 4.6. Therapeutic Ultrasound

Therapeutic ultrasound provides energy in the form of sound, which is absorbed by tissues of high protein content such as skeletal muscle, thereby providing deep heating to target tissues. Short-term (<10 minutes) heating of 1.6–4.6 degrees Celsius was reported in the caudal thigh muscles of dogs following ultrasound, with results dependent on the selected power (1–1.5 W/cm^2^). Calcaneal tendon extensibility and tarsal flexion also increased for 5 minutes following a similar protocol in dogs [44]. The short-term effects of ultrasound are likely most beneficial when followed by range of motion exercises. Repeated and frequent administration may produce faster rates of healing in tendons and in other tissues, but this approach is often constrained by logistical and financial considerations in clinical practice [45]. The facilitation of tissue stretching and thermal heating was used more often in orthopedic as opposed to neurologic patients, likely due to the more severe range of motion restrictions noted in many orthopedic conditions, such as cranial cruciate ligament rupture [15].

### 4.7. Massage Therapy

Massage therapy may have similar effects as to the aforementioned modalities. The techniques and goals of such manipulations are similar to those described in humans, although there is minimal scientific literature regarding the efficacy of massage in dogs and cats [46]. The principles by which massage might benefit an integrative medicine patient, based on available human studies, include increased lymphatic flow, modulation of local pain mediators such as substance P and prostaglandins, positive cortical responses, increased local circulation, reductions in muscle spasms and pain, and reduction of tissue adhesions [46]. Massage was the least frequently applied therapeutic modality, which may be related to the time required for the treatment or to the perception that manual therapies are less effective than other interventions. The latter cannot be determined until further veterinary studies are performed.

### 4.8. Herbal Interventions

Herbal recommendations were made to a small number of clients for a number of different conditions. A discussion of the merits of herbal supplements is outside the scope of this investigation, but there are a number of herbs and herbal formulas suggested to impart a clinical effect in veterinary species [47, 48]. Unfortunately, herbal veterinary products have minimal regulatory oversight, as neither the Association of American Feed Control Officials (AAFCO) nor the United States Food and Drug Administration (FDA) controls these products. A previous study of Chinese herbal formulas identified variable amounts of minerals and potential contaminants, and some contain small amounts of potentially toxic compounds [49, 50]. The herbal recommendations in this study were made by the supervising clinician based on a review of the conventional therapies and with a risk-benefit discussion with the owner, recognizing that interactions with drugs or unpredictable reactions are of real concern [51].

### 4.9. Nutritional Recommendations and Obesity Prevalence

Nutritional recommendations were provided to the owners in approximately 15% of cases. This is in contrast to current recommendations that every patient receives a nutritional assessment and recommendation [52]. All audited patient records except for one contained a weight and body condition score, and the multiple modality visits were nearly all recheck examinations. Therefore, it is likely that the collected data underestimates the percentage of patients receiving nutritional recommendations in the service. The importance of communication is stressed in current consensus guidelines and as such a revision of the patient discharge form to reinforce nutritional plans and monitoring appears warranted.

More than half of the dogs treated were overweight or obese. This prevalence is consistent with a recent study in the United Kingdom but higher than a dated study performed in the United States [53, 54]. Nevertheless, the animals in this study do not appear to be more overweight than the general population. A limitation of the collected retrospective data is that the same clinician did not perform all body condition scoring, but a validated nine-point system was available to all service clinicians. Dachshunds and Labrador retrievers were both more likely to be overweight than were other breeds in this study. A previous study identified that both breeds had increased risk of being overweight (OR = 1.6) and that Dachshunds also displayed increased risk for obesity (OR = 1.7) [53]. An association between body condition score and the number of visits or the presenting complaint was not detected, suggesting either that body condition did not contribute to the observed pathologies or, more likely, that excess adiposity contributed equally to the patients' conditions.

### 4.10. Breed, Age, and Gender Distribution of the Study Population

The breed distributions observed in this study are remarkably similar to a previous survey of general breed prevalence [55]. Mixed breeds accounted for 27% of this patient population and the same amount was reported in a national survey. Labrador retrievers comprised 7.4% and German Shepherds 3.3% which is again similar to the 7.9% and 3%, respectively, reported in the previous population study. Therefore, only the Dachshund appears overrepresented when compared to available data; they were 15.2% of the treated dogs, which dramatically contrasts with published statistics that they are less than 1.5% of the general population.

The predisposition of Labradors to orthopedic disease in this study is consistent with previous reports [56, 57], as is the predisposition of Dachshunds to intervertebral disc disease [57, 58]. The Dachshund accounted for 31.8% of all cases with a primary neurologic complaint, and a previous report found that Dachshunds comprised 48.0% of all canine intervertebral disc disease cases [58]. In the present study, intervertebral disc disease was included in a broader category of all neurologic disease and therefore a definitive comparison between the reported prevalence data cannot be made. The average age of the treated Dachshunds (7.9 years) is older than the age of highest risk for IVDD in chondrodystrophic breeds of 4–6 years [58], which could represent an anomaly. The finding could also suggest that older Dachshunds are more likely to require rehabilitation or those owners are more likely to elect therapy for this population.

Treated dogs were older (median = 9) and more likely to be neutered when compared to general population statistics (median age = 4.8) [55]. Intact males comprised 8.5% of caseload, neutered males 47%, intact females 4%, and spayed females 40.5% as compared to 21.2%, 24.4%, 16.7%, and 37.1%, respectively. It is unclear if neutering is causally related to the orthopedic and neurologic diseases treated in this study although an association between early neutering and joint disorders has been suggested [59].

### 4.11. Treatment Differences between Orthopedic and Neurologic Patients

The present data demonstrates that more than 15% of patients presenting with orthopedic or neurologic disease will have concurrent pathology of the other type. Therefore, a thorough physical examination and inventory of all conditions should be performed in all integrative medicine patients. Given that neurologic and orthopedic patients received the same number of modalities, it is logical to suggest that the same amount of time can be dedicated to orthopedic and neurologic cases. However, orthopedic patients were treated with more sessions during the study period than were those with neurologic disease. It could be hypothesized that this is because some neurologic patients, particularly those with intervertebral disc disease, make a full and complete recovery whereas many orthopedic diseases are chronic. Identification of overweight and obese patients is equally important in both groups as neither was more predisposed to obesity than the other.

### 4.12. Study Limitations

The study is subject to several limitations. The medical record system did not allow for clear examination of efficacy across the range of conditions that were treated. Prospective studies of each modality are needed. As is true of most individualized patient rehabilitation protocols, the effectiveness of a combination protocol, as opposed to a single modality treatment, was not assessed. The combination of modalities selected by a clinician could act synergistically or could negate the benefits of other therapies. The reason for selection of modalities was not explored, and the bias of the different service clinicians may confound the presented results. Additionally, detailed data is not presented on other types of appointments although information on hyperbaric oxygen treatments performed by the service is available elsewhere [60].

The data are heavily skewed toward dogs, but this is not surprising given that more of the small animal publications in integrative medicine, especially rehabilitation, focus on dogs. Additional investigation is warranted in practices that support a higher feline caseload. Similarly, equine cases were insufficient to be compared to dogs and are likely different in the modalities elected and the patient demographics. A limitation of this study is that detailed data was only collected from one treatment sheet from each patient's record, and therefore the audited data may not accurately reflect the characteristics of the patients despite randomization. However, the large volume of cases prohibited a more comprehensive analysis.

### 4.13. Controversies in Integrative Veterinary Medicine

General controversies regarding the efficacy and suitability of integrative medical practice exist, and this study may incite criticisms of the use of these modalities in integrative practice. The amount of evidence for each technique employed in this study is admittedly unknown. One prominent integrative medicine scholar in the human medical field has proposed that only 7.4% of complementary and alternative techniques have compelling evidence justifying their use [61]. No similar attempt has been made to characterize the evidence for integrative or conventional veterinary therapies. The veterinary profession has very few interventions which reach the highest evidence grade, and significant challenges of the application of evidence-based medicine to veterinary practice have been described [62]. These include the expense of randomized controlled trials, the reliance on retrospective studies, the smaller population of researchers and research funding, the inherent difficulties in searching available databases, and the veterinarian's reliance on clinical experience. Clearly, however, integrative veterinary medicine should adopt the recommendations of McKenzie that all interventions, whether integrative, complementary, alternative, or holistic, be subjected to the same investigative standards and that practitioners accept both the primacy of empirical evidence and willingness to modify their practice based on such evidence [63]. This conversely requires skeptics of integrative medicine to support evidence-based integrative medicine based on the available data and evidence rather than on a perceived scale of acceptability. The authors of this paper hope that the information contained herein can provide a starting point for continued research on the applicability of these and other modalities.

## 5. Conclusions

The collected data provide a foundation for future prospective evaluations of integrative veterinary medicine. The prevalence of acupuncture, laser, and hydrotherapy in this patient population suggests that these should be initial areas of research. Older dogs with neurologic or orthopedic diseases were the primary patient populations treated, and Dachshunds were overrepresented compared to national statistics. Therefore, these patients should likely serve as the target populations for additional study. The data further support that integrative medicine can be successfully incorporated into conventional multispecialty referral practices with a high degree of cooperation. Additional evidence-based outcome measures should be used to evaluate the efficacy of integrative therapies, and both veterinarians and veterinary students should be prepared to counsel owners in these areas.
